# Supplementary material for: Microbial Transformations of Halolactones and Evaluation of Their Antiproliferative Activity
Source: Int J Mol Sci. 2023 Apr 20;24(8):7587. doi: 10.3390/ijms24087587 (PMC10144491; doi:10.3390/ijms24087587)
Supplement: Supplementary file 1 [file ijms-24-07587-s001.zip › ijms-2357539-supplementary.pdf]

International Journal of Molecular Sciences

Supplementary Materials

Microbial transformations of halolactones and evaluation of their antiproliferative activity

Marcelina Mazur[1],*, Karolina Maria Zych[1], Bożena Obmińska-Mrukowicz[2], Aleksandra Pawlak[2]

[1]Department of Food Chemistry and Biocatalysis, Wrocław University of Environmental and Life Sciences, Norwida 25, 50-375 Wrocław, Poland

[2] Department of Pharmacology and Toxicology, Wrocław University of Environmental and Life Sciences, C.K. Norwida 31, 50-375 Wrocław, Poland

*Correspondence: Dr. Marcelina Mazur marcelina.mazur@upwr.edu.pl; Tel/Fax.: +48-713205197

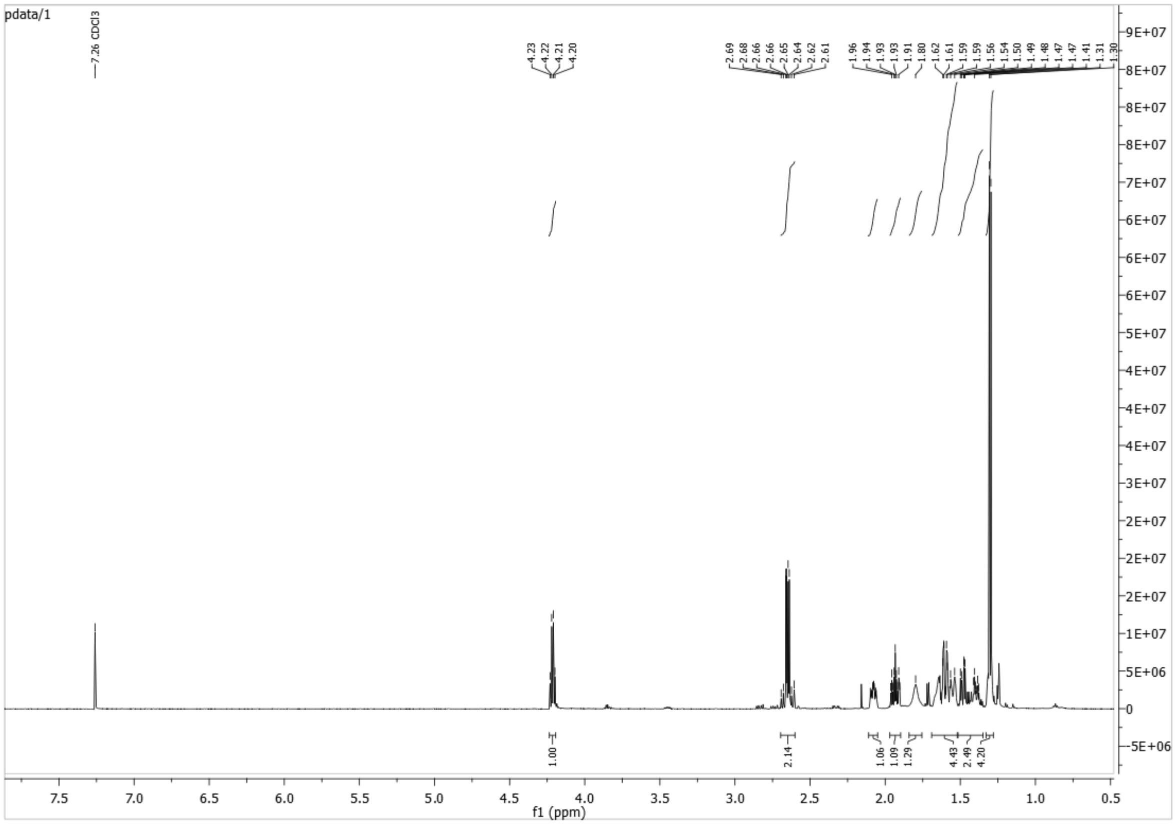

Figure S1: ^{1}H-NMR spectrum of hydroxylactone 8.

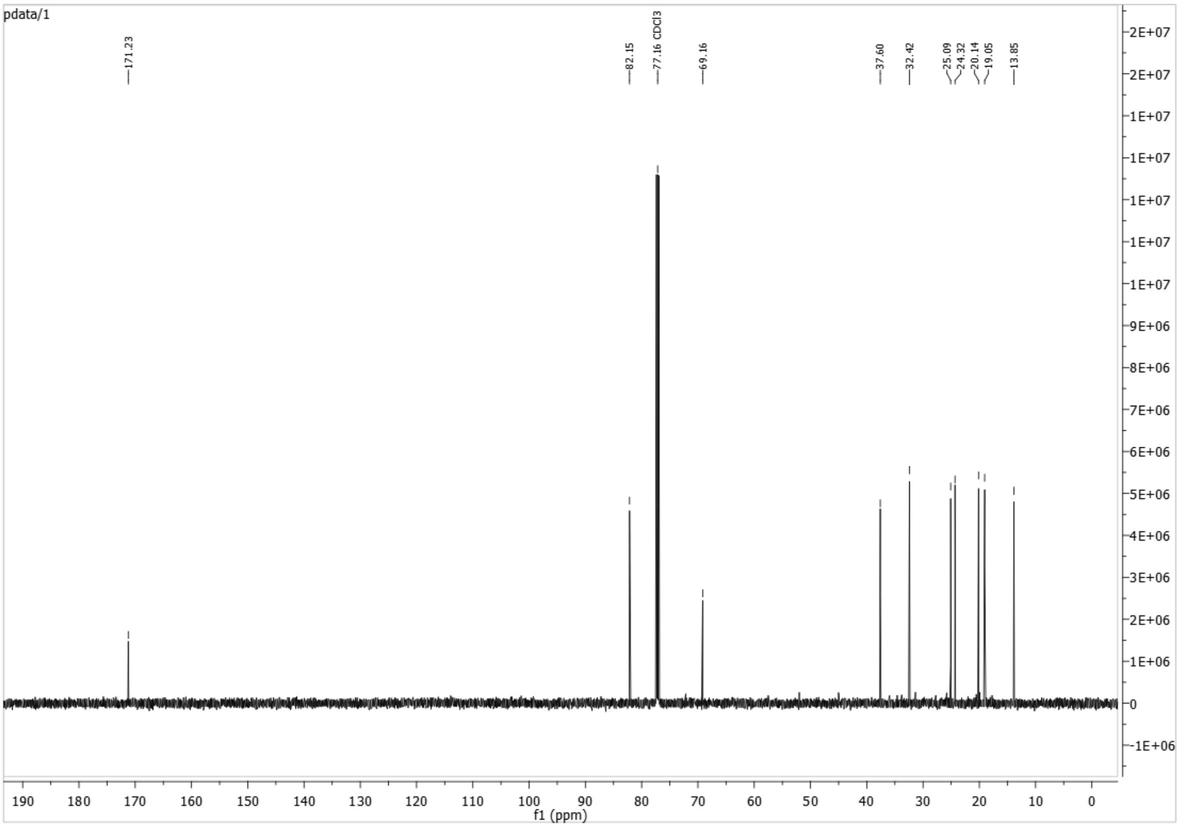

Figure S2: ^{13}C-NMR spectrum of hydroxylactone 8.

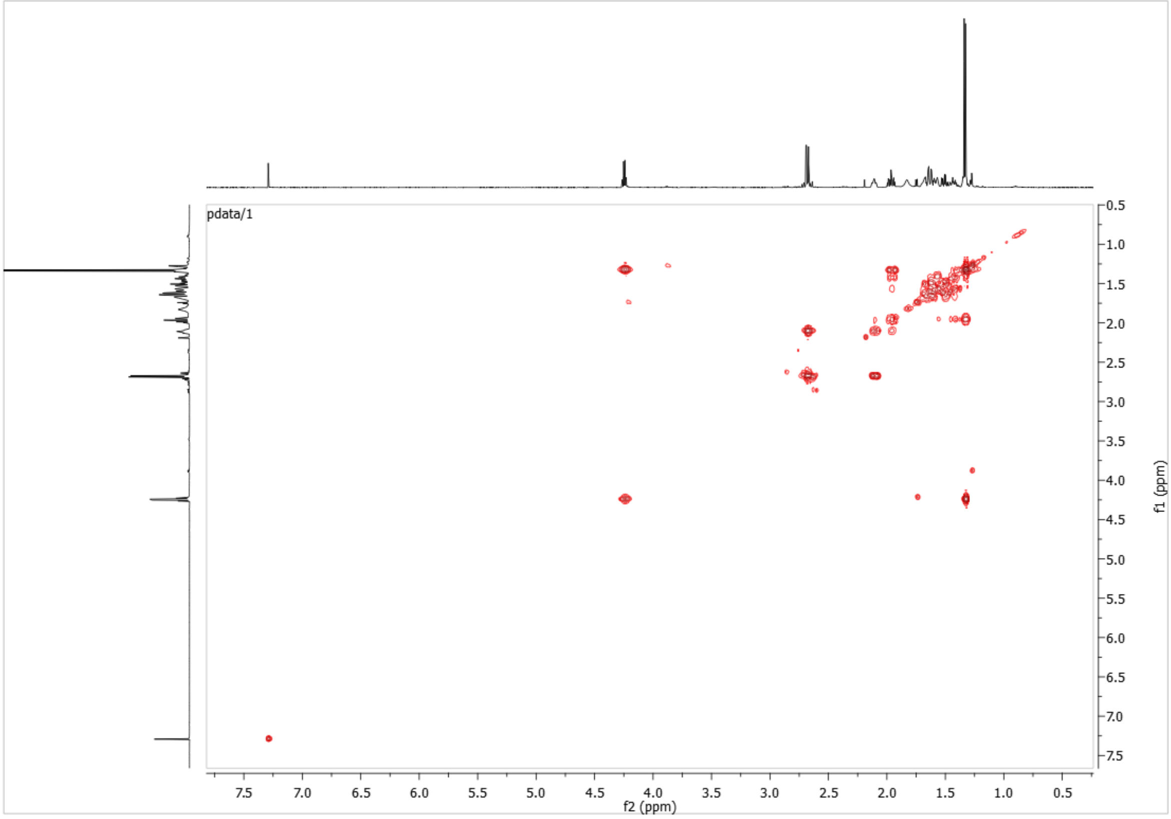

Figure S3: COSY spectrum of hydroxylactone 8.

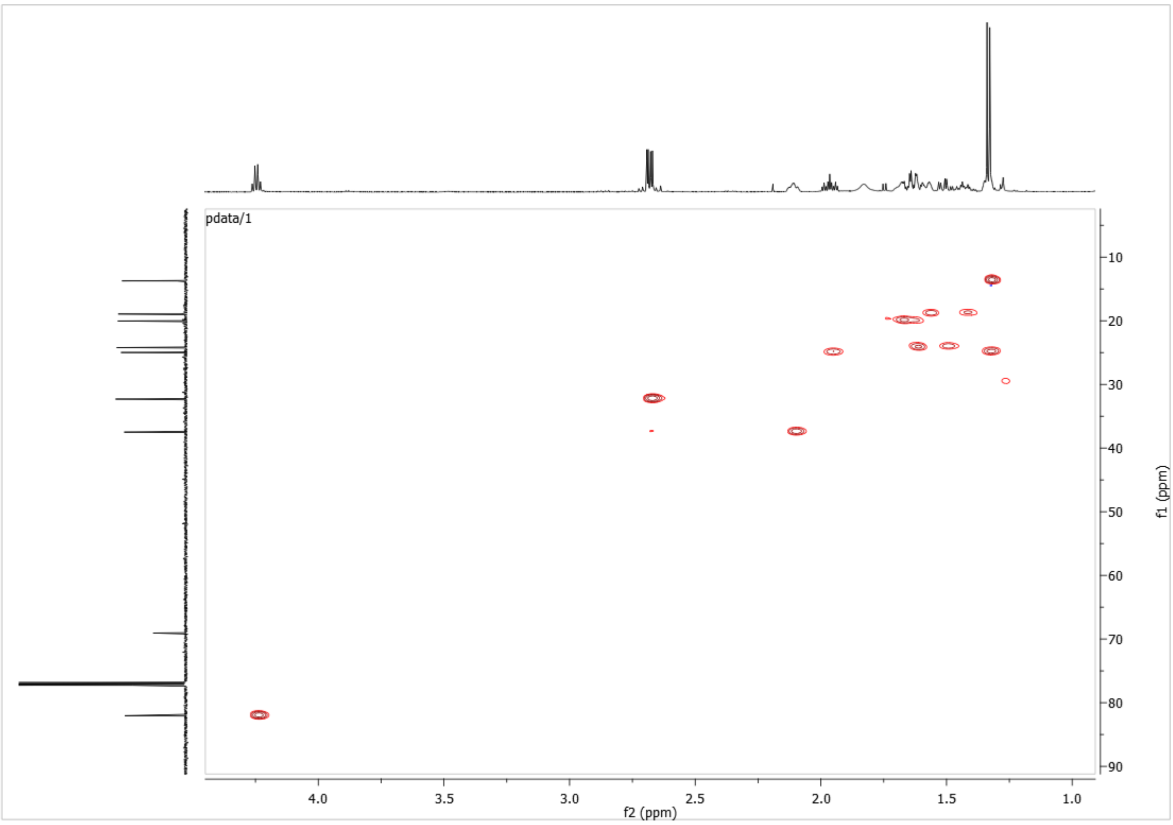

Figure S4: HSQC spectrum of hydroxylactone 8.

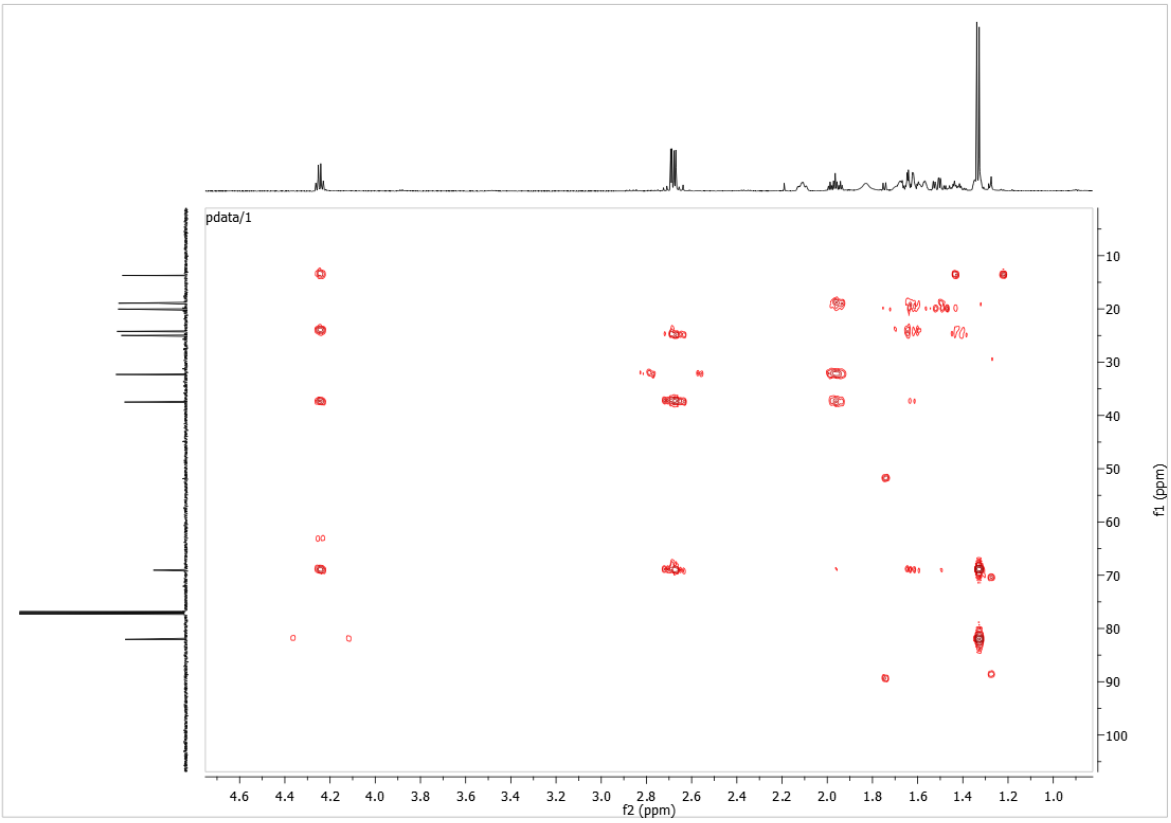

Figure S5: HMBC spectrum of hydroxylactone 8.

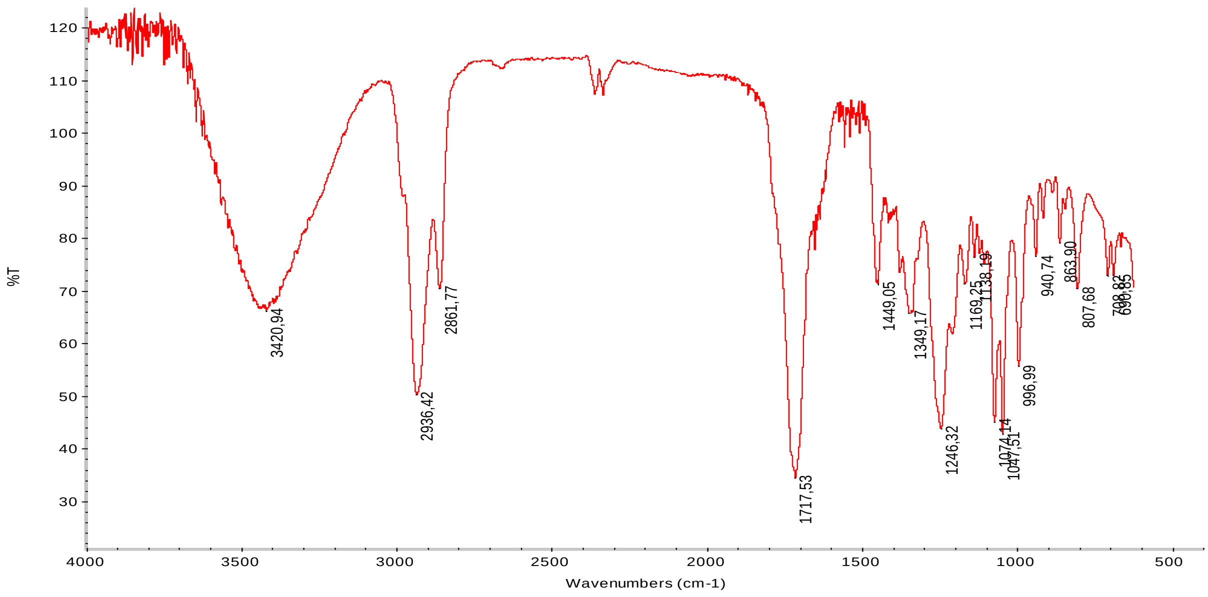

Figure S6: IR spectrum of hydroxylactone 8.

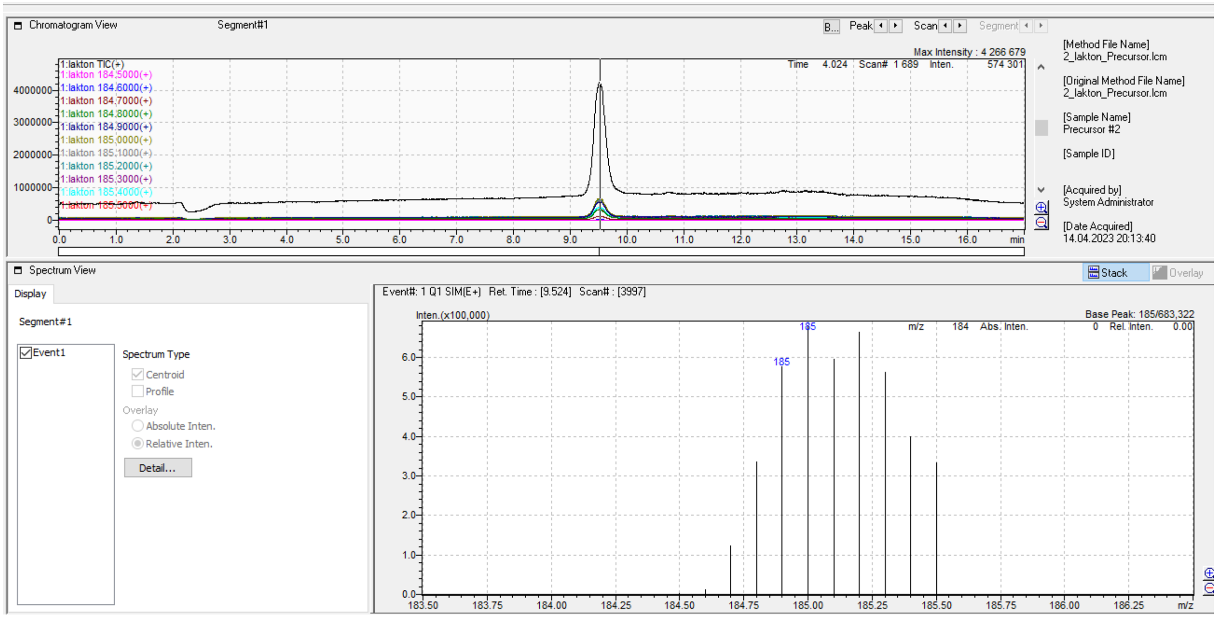

Figure S7: ESISMS analysis of hydroxylactone 8.
